# Factors Associated With Cancer Disparities Among Low-, Medium-, and High-Income US Counties

**DOI:** 10.1001/jamanetworkopen.2018.3146

**Published:** 2018-10-05

**Authors:** Jeremy M. O’Connor, Tannaz Sedghi, Meera Dhodapkar, Michael J. Kane, Cary P. Gross

**Affiliations:** 1 Yale University School of Medicine, New Haven, Connecticut; 2 National Clinician Scholars Program, New Haven, Connecticut; 3 Cancer Outcomes, Public Policy and Effectiveness Research Center, Yale University School of Medicine, New Haven, Connecticut; 4 The University of Chicago, Chicago, Illinois; 5 Yale University School of Public Health, New Haven, Connecticut

## Abstract

**Background:**

There are concerns about the degree to which county income level might underlie the stark disparities in cancer death rates among US counties; at the same time, there is uncertainty about the factors that may mediate the disparities.

**Objectives:**

To assess county-level cancer death rates and to identify possible mediators of the association between county-level median incomes and cancer death rates.

**Design, Setting, and Participants:**

Cross-sectional study using death records from the National Center for Health Statistics in 2014, with data collected and analyzed between October 1, 2016, and July 31, 2017. All US counties and county equivalents were included.

**Exposures:**

County-level median household income.

**Main Outcomes and Measures:**

County-level age-standardized cancer death rate.

**Results:**

In 3135 counties, median incomes ranged from $22 126 to $121 250 per year. Low-income counties (median income, $33 445) vs high-income counties (median income, $55 780) had higher proportions of residents who were non-Hispanic black, lived in rural areas, or reported poor or fair health. The mean (SD) cancer death rate was 185.9 (24.4) per 100 000 person-years in high-income counties, compared with 204.9 (26.3) and 229.7 (32.9) per 100 000 person-years in medium- and low-income counties, respectively. In mediation models, health risk behaviors (smoking, obesity, and physical inactivity); clinical care factors (unaffordable care and low-quality care); health environments (food insecurity); and health policies (state smoke-free laws and Medicaid payment rates) in aggregate accounted for more than 80% of the income-related disparity. The strongest possible mediators were food insecurity (explaining 19.1% [95% CI, 12.5%-26.5%] of the association between county incomes and cancer deaths), low-quality care (17.9%; 95% CI, 14.0%-21.8%), smoking (12.7%; 95% CI, 9.4%-15.6%), and physical inactivity (12.2%; 95% CI, 9.4%-15.6%).

**Conclusions and Relevance:**

There are wide gaps in cancer death rates between low-, medium-, and high-income counties. Future studies are needed to assess whether targeting the possible mediators might ameliorate the substantial socioeconomic cancer disparities.

## Key Points

**Question:**

How substantial are cancer disparities on the basis of county levels of income, and what are the factors that may mediate the disparities?

**Findings:**

In this cross-sectional study of 3135 US counties, cancer death rates varied significantly in counties of different income levels, with a mean cancer death rate per 100 000 person-years of 185.9 in high-income counties, 204.9 in medium-income counties, and 229.7 in low-income counties. The strongest possible mediators were health risk behaviors, cost and quality of clinical care, and food insecurity.

**Meaning:**

There are multiple county-level factors that may serve as mediators of cancer disparities and that may be targeted by future efforts to achieve equity in cancer outcomes.

## Teaser

This cross-sectional study uses death records from the US National Center for Health Statistics to investigate the association between cancer death rates and county-level median household income and identifies potential mediating factors of this association.

## Introduction

Advances in cancer prevention, diagnosis, and treatment have led to rapid reductions in cancer mortality in the United States, with cancer death rates decreasing from 240 per 100 000 person-years in 1980 to 192 per 100 000 person-years in 2014.^1,2,3^ Reductions in cancer mortality, however, are not synonymous with reductions in cancer disparities—perhaps in part because of variation in access to advances in care. Indeed, cancer disparities remain substantial in the United States by geographic area and by socioeconomic status.^4,5,6^ The socioeconomic cancer disparities are notable in particular because they may worsen over time owing to the soaring costs of cancer diagnosis and treatment. Thus, there are major concerns about US socioeconomic disparities in cancer deaths.

A second major concern is growing evidence of wide disparities in cancer mortality at the county level, with cancer death rates that varied more than 7-fold across US counties in 2014.^2^ At the same time, there is a gap in knowledge about the degree to which socioeconomic factors might underlie the county disparities, or more specifically, the clustering of counties into area hot spots with a disproportionate burden of cancer deaths. Identifying area hot spots can be useful as a guide for public health programs to target the neediest clusters of counties.^3^ The success of such programs, however, depends on understanding the factors that underlie the disparities.

To reduce cancer disparities, it is imperative to understand the degree to which multiple environmental, clinical, and behavioral factors may serve as mediators of the association between county income and cancer mortality.^3,7,8,9,10^ There are gaps in knowledge, however, about these key factors. First, it is unclear which of the factors are serving as the strongest mediators of the association between socioeconomic status and cancer mortality. Second, it is unclear whether such factors adequately explain the large differences in cancer mortality between counties. This is important in part because a better understanding of the possible mediators of high death rates among low-income counties is needed to inform future efforts to lessen disparities.

In this context, we studied the association between county-level incomes and cancer death rates. Our aims were to assess the disparities in cancer death rates between low-, medium-, and high-income counties; to assess geographic variation in cancer death rates within and between income groups; to identify factors that may serve as mediators of county disparities; and finally, to compare geographic variations in associated factors with geographic variations in cancer deaths.

## Methods

### Study Design

We conducted a cross-sectional study to assess cancer disparities between counties on the basis of socioeconomic status and to identify clusters of counties with high cancer death rates. Then we used regression models to identify multiple factors associated with the disparities and to test our hypothesis that these factors may serve as mediators of disparities between counties. Finally, to assess geographic variation in possible mediators across counties, we used the sums of the values of the possible mediators to calculate a standardized risk score that we called the disparity risk index. The Yale institutional review board determined this study was exempt from review because we used publicly available data. This study followed the Strengthening the Reporting of Observational Studies in Epidemiology (STROBE) reporting guideline.

### Data Sources

We included all counties with cancer death rates available from the year 2014 in a database the Institute for Health Metrics and Evaluation created and published.^11^ To create the database, institute researchers used death record data from the National Center for Health Statistics to generate small area estimates and to eliminate garbage codes, which are implausible or nonspecific causes of death.^12^ As noted in prior studies, this approach is likely to improve the validity of county-level death records as key metrics for identifying specific causes of death.^2,12^

We linked the cancer death rate of each county to the median household income (MHI). We used income data from the 2012 US Census Bureau Small Area Income and Poverty Estimates, thus allowing for a 2-year lag prior to our primary outcome measure from 2014.^13^ We used MHI instead of using employment, education, or a combined socioeconomic indicator because MHI is a widely used, readily available marker of socioeconomic status,^14,15,16^ and because we expected it to have the most relevance to cancer death rates because of the high financial burden of cancer care.

### Outcomes, Exposures, and Possible Mediators

Our primary outcome was the age-adjusted cancer death rate per 100 000 person-years. To identify the factors that may serve as mediators of the association between the exposure and the outcome at the county level, we used time-lagged variables from the Robert Wood Johnson Foundation County Health Rankings conceptual model (eTable 1 in the Supplement). This model systematically evaluates and ranks counties according to a series of health risk factors that are selected on the basis of their validity and their importance in public health.^17,18^ It then groups these health factors into domains: health risk behaviors, clinical care factors, socioeconomic factors, and physical environment factors.^18^ We added a fifth domain to include cancer-relevant health policies—for example, the number of state-level mandates for insurance coverage of cancer care. Last, we added other factors, such as the presence of a comprehensive cancer center nearby, that were not included in the County Health Rankings model but were expected to be relevant to cancer outcomes.^19,20^ We obtained factors from multiple sources, including the Centers for Disease Control and Prevention, the American Lung Association, the National Cancer Institute, and the American Society of Clinical Oncology (eTable 1 in the Supplement).^21,22,23,24,25,26^

### Statistical Analysis

We stratified counties into low- (lowest quartile), medium- (middle quartiles), and high-income (highest quartile) groups. We used descriptive statistics to determine the mean cancer death rates and the demographic characteristics.

**Figure 1.**
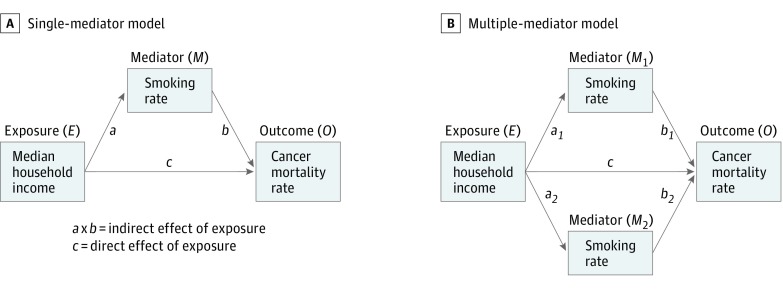
Schema for Models of Mediation We adapted schemas for single-mediator (A) and multiple-mediator (B) models from Preacher and Hayes.^27^ The subsequent models of mediation can be used to estimate the direct effects of *E* on *O* and the indirect effects of *E* on *O* that might be attributable to *M*. The subscripts in panel B identify the separate pathways for the multiple mediators (ie, *M_1_* vs *M_2_*).

To identify the factors that account for the county disparities, we fit a series of models of possible mediators (eTable 1 in the Supplement includes a list of variables). Mediators are helpful in modeling pathways between exposures and outcomes, as shown in Figure 1.^27^ Furthermore, models can simplify complex pathways by separating direct effects, which are independent of the mediator, from indirect effects, which are dependent on the mediator. In this way, we used the models to identify the intermediary factors (mediators) that help to explain the association between MHI (the exposure) and cancer mortality (the outcome).

To quantify the associations, we used a series of linear regression models. First, we used single-mediator models to assess for changes in parameter estimates after adding possible mediators to a base model of the outcome (cancer death rate, a continuous variable) and the exposure (MHI, a continuous variable that we log transformed).^28^ We selected variables having significant associations in the single mediator models (2-sided α < .05) to be tested in the multiple mediator model. We used backward stepwise elimination to retain the variables that remained significant in the multivariable model. We adjusted for demographic factors including racial and ethnic distributions of county residents (eTable 1 in the Supplement) and we compared the stepwise approach with other approaches (eg, least absolute shrinkage and selection operator) in sensitivity analyses. After identifying the possible mediators, we estimated the indirect effects, which are given by the product of the coefficients of the association between (1) the exposure and the mediators and (2) the mediators and the outcome.^27^ We used a method called *seemingly unrelated regression,* which is used to correct for the possibility of correlations between error terms in a series of similar models.^29^ We used the indirect effects to calculate the percentage mediated (ie, the percentage of the exposure-outcome association that can be explained by the possible mediator) and we used bootstrapped standard errors with 5000 repetitions because the errors for indirect effects are often skewed.^27^

We used multivariate normal regression to impute 20 sets of values for variables with greater than 5% but less than 20% missingness and excluded variables with greater than 20% missingness (eTable 2 in the Supplement). In sensitivity analyses, we compared models with or without outlier counties (for example, those with very large or small populations or with >40% Native American ethnicity). In addition, because of concerns in prior studies about counties with much higher than expected rates of mortality (for example, rates being more than 3 times the interquartile range beyond the quartile values),^30^ we performed sensitivity analyses that excluded these counties.

### Calculating the Disparity Risk Index

To assess the geographic distribution of the possible mediators, we calculated a composite score that we called the *disparity risk index* (eFigure 1 in the Supplement). Each county’s value for the risk index was the weighted sum of the variables’ standardized values (or *z* scores). Thus, we used the risk index to standardize the comparisons of variables across counties.

To assess the geographic variation in the outcome, we mapped the rates of cancer deaths using geocoded templates. We identified area hot spots using the Getis-Ord statistic for spatial autocorrelation with a significance threshold of 2-sided *P* < .05.^31,32^ We then mapped the possible mediators (as represented by the disparity risk index) and we identified and then mapped the hot spots for each of the possible mediators. We completed the analysis between October 1, 2016, and July 31, 2017, using Stata statistical software version 14.2 (StataCorp LLC).

## Results

In our total sample of 3135 US counties, the median incomes ranged from $22 126 to $121 250 per year. Compared with counties in the high-income group (median income, $55 780), those in the low-income group (median income, $33 445) had smaller populations in addition to having higher proportions of residents who were non-Hispanic black, lived in rural areas, or reported poor or fair health (Table 1). Cancer death rates varied widely across counties (mean [range] rate, 206.4 [70.7-503.5] deaths per 100 000 person-years).

**Table 1.** Characteristics of US Counties Stratified by Median Household Income

Characteristics	County-Level Income Groups, Mean (SD)		
	Low (n = 783)	Medium (n = 1568)	High (n = 784)
Household income, median (IQR), $	33 445 (31 253-35 483)	43 010 (40 298-46 306)	55 780 (52 409-63 376)
Population, median (IQR), No.	16 996 (9474-28 543)	26 932 (10 659-62 800)	59 227 (17 417-187 729)
Age ≥65 y, %	17.4 (4.0)	17.5 (4.4)	14.6 (3.8)
Female, %	50.0 (2.9)	50.0 (2.1)	50.1 (1.8)
Race, %			
Non-Hispanic white	68.3 (24.8)	81.9 (16.7)	80.2 (16.5)
Non-Hispanic black	18.0 (21.4)	6.0 (9.9)	5.3 (7.9)
Hispanic	8.8 (17.4)	8.1 (12.1)	9.0 (10.5)
Asian	0.5 (0.6)	0.9 (1.3)	2.7 (4.6)
Native American	3.4 (11.8)	1.9 (5.6)	1.4 (4.4)
Rural, %	72.4 (25.7)	59.2 (30.2)	43.6 (32.5)
Poverty (income <100% federal poverty level), %	24.2 (5.9)	16.2 (4.0)	10.8 (3.3)
Premature deaths per 100 000 person-years, No.^a^	489.3 (96.7)	382.4 (72.7)	310.6 (61.8)
Reported poor or fair health, %	23.3 (5.8)	16.5 (4.8)	12.8 (3.7)
Poor physical health, %	4.7 (1.2)	3.7 (1.0)	3.2 (0.7)
Cancer deaths per 100 000 person-years, No.^a^	229.7 (32.9)	204.9 (26.3)	185.9 (24.4)

Abbreviation: IQR, interquartile range.

^a^ Age adjusted.

**Figure 2.**
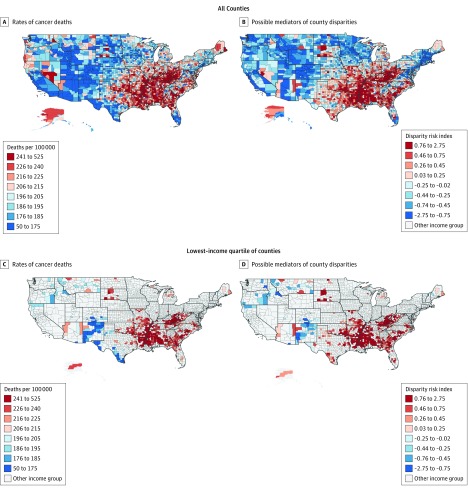
County Rates of Cancer Deaths and Potential Mediators of Disparities in Cancer Death Rates Between Low-, Medium-, and High-Income US Counties A, Age-adjusted annual cancer death rate. B and D, Values for the disparity risk index, which we used to represent the geographic spread of the key factors that may act as mediators of county income-related disparities in cancer death rates. C, Income-adjusted annual cancer death rate in each county for the lowest-income quartile of counties.

We found significant variation in cancer death rates across income groups, with a mean (SD) rate of 229.7 (32.9) deaths per 100 000 person-years in low-income counties vs 204.9 (26.3) (difference, 24.8; 95% CI, 22.4-27.4) and 185.9 (24.4) (difference, 43.8; 95% CI, 41.0-46.7) per 100 000 person-years in medium- and high-income counties, respectively (*P* < .001 for all pairwise comparisons). We found geographic clusters, or hot spots, with the highest cancer death rates in the South, including the Mississippi River Delta, in addition to Appalachia (n = 507 counties in hot spots at a threshold of *P* < .05) (Figure 2; eFigure 2 in the Supplement). Many of these hot spots were constituted with low-income counties (Figure 2).

Of the 38 county-level factors we assessed in this study, 19 met criteria for inclusion in the multiple-mediator model, and 8 retained significance in the fully adjusted model, suggesting that 8 county-level factors may be serving as mediators of county-level socioeconomic cancer disparities (Table 2). These factors are important because they correlate with the exposure (county-level MHI) and the outcome (cancer death rate) and because they might fit plausibly into the relevant causal pathways. Three of these factors were health risk behaviors (rates of obesity, smoking, and physical inactivity); 2 were clinical care factors (indicators of unaffordable care and low-quality care); 2 were health policies (smoke-free laws and the state Medicaid fee index, which is a state-level ratio of provider payments from Medicaid vs Medicare); and 1 was a health environment factor (food insecurity, defined as the percentage of the population that lacks a reliable source of food). In aggregate, these factors explained more than four-fifths (81.25%) of the association between county-level median incomes and cancer death rates.

**Table 2.** Potential Mediators of the Association Between County Median Incomes and Cancer Death Rates Identified by Multiple Mediator Model

Independent Variables^a^	β Coefficient (95% CI)	Percentage Mediated^b^
**Direct Associations–Possible Mediator Variables**		
Health behaviors		
Obesity	1.41 (1.15 to 1.66)	NA
Smoking	0.80 (0.63 to 0.98)	NA
Physical inactivity	0.90 (0.66 to 1.13)	NA
Clinical care		
Unaffordable care	0.34 (0.17 to 0.52)	NA
Low-quality care	2.22 (1.90 to 2.55)	NA
Health policies		
Smoke-free laws	−0.33 (−0.49 to −0.17)	NA
Medicaid-Medicare fee index	−0.15 (−0.20 to −0.10)	NA
Health environment		
Food insecurity	1.12 (0.76 to 1.47)	NA
**Direct Association–Primary Exposure Variable**		
Median household income	−0.12 (−0.18 to −0.07)	NA
**Indirect Associations–Possible Mediator Variables**^c^		
Health behaviors		
Obesity	−0.07 (−0.09 to −0.05)	10.8
Smoking	−0.08 (−0.10 to −0.06)	12.7
Physical inactivity	−0.08 (−0.10 to −0.06)	12.2
Clinical care		
Unaffordable care	−0.03 (−0.05 to −0.02)	5.2
Low-quality Care	−0.11 (−0.14 to −0.09)	17.9
Health policies		
Smoke-free laws	−0.01 (−0.01 to −0.003)	1.1
Medicaid-Medicare fee index	−0.01 (−0.02 to −0.01)	1.7
Health environment		
Food insecurity	−0.12 (−0.17 to −0.08)	19.1
Total indirect (mediated)	−0.52 (−0.58 to −0.46)	81.3^d^

Abbreviation: NA, not applicable.

^a^ We included the following county demographic factors as independent variables in the model because we expected them to be potential confounders: the racial/ethnic distributions of residents, the percentage of the county that is rural, the percentage female residents, and the percentage non-English-speaking residents. All *P* values are <.001 except for *P* = .002 for the indirect association with smoke-free laws.

^b^ The percentage mediated is the percentage of the total exposure-outcome association that is attributed to the possible mediator. The numerator is the coefficient for the indirect (mediator-outcome) association, and the denominator is the sum of the coefficients (direct and total indirect associations) for the exposure-outcome association.

^c^ Indirect associations are the subsets of the total exposure-outcome association attributed to each mediator. In contrast, direct associations are mediator-outcome associations that are independent from the exposure.

^d^ Total values may differ slightly from the sum of the reported values because of rounding.

**Figure 3.**
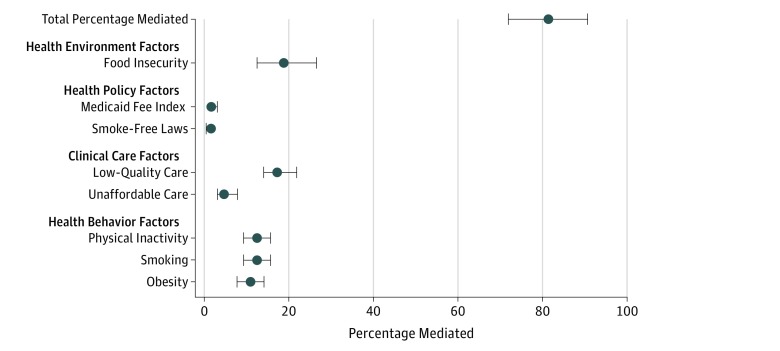
Percentage Mediated by County-Level Factors in a Multivariable Model of the Association Between County-Level Median Household Incomes and Cancer Death Rates For the definitions and the sources of the data for the factors that may serve as mediators, see eTable 1 in the Supplement. To calculate the percentage mediated, we used the β coefficients from the multiple mediator model reported in Table 2. Error bars represent 95% CIs.

We found substantial variation in the degree to which each of the factors may be mediating the county-level association between incomes and death rates. The strongest possible mediators were food insecurity (β = −0.12; 95% CI, −0.17 to −0.08), low-quality care (β = −0.11; 95% CI, −0.14 to −0.09), smoking (β = −0.08; 95% CI, −0.10 to −0.06), physical inactivity (β = −0.08; 95% CI, −0.10 to −0.06), and obesity (β = −0.07; 95% CI, −0.09 to −0.05) (Table 2). Per the final model, the percentage mediated may be largest for food insecurity (19.1%; 95% CI, 12.5%-26.5%), low-quality care (17.9%; 95% CI, 14.0%-21.8%), smoking (12.7%; 95% CI, 9.4%-15.6%), and physical inactivity (12.2%; 95% CI, 9.4%-15.6%), and it may be smallest for smoke-free laws (1.1%; 95% CI, 0.5%-1.6%) and the Medicaid fee index (1.7% 95% CI, 1.6%-3.1%) (Figure 3).

We summarized the geographic spread of the factors using the disparity risk index. We found stark differences in the index between low-income (mean [SD], 0.64 [0.57]), medium-income (mean [SD], 0.03 [0.48]), and high-income (mean [SD], −0.58 [0.50]) counties, with higher scores indicating higher concentrations of possible mediators. In models adjusted for county income, the disparity index was associated with cancer death rates overall (β = 35.7; 95% CI, 34.2-37.2; *P* < .001) and in the subgroup of low-income counties (β = 39.5; 95% CI, 36.2-42.8; *P* < .001). We used maps of the index to visualize whether groups of low-income counties having high concentrations of possible mediators matched with groups of low-income counties having high concentrations of cancer deaths (Figure 2). In addition, we identified hot spots of adjacent counties with similarly high values for each of the possible mediators, which included 391 counties in hot spots for smoking; 361, obesity; 469, physical inactivity; 391, food insecurity; 255, the fee index; 468, smoke-free laws; 315, unaffordable care; and 358, low-quality care (all *P* < .05) (eFigure 2 in the Supplement).

In sensitivity analysis, we found similar estimates in models with or without counties that were outliers in demographic characteristics or cancer death rates. In addition, other approaches to variable selection (eg, least absolute shrinkage and selection operator) led to identical models. Thus, the final multivariable model seemed to provide an accurate and robust estimate of the degree to which the factors may explain the disparities.

## Discussion

We found substantial socioeconomic disparities in cancer death rates across US counties and identified the factors that may account for more than 80% of the county-level disparity. By creating a disparity risk index, a composite measure of the factors that may mediate the association between county levels of income and cancer death rates, we identified heterogeneity in the geographic spread of the factors that aligned with the distribution of cancer death rates in low-income counties. Together, these findings suggest there are multiple factors in low-income areas that might be useful targets for actions to ameliorate cancer disparities between counties.

Our findings are consistent with prior studies of cancer disparities by area markers of socioeconomic status,^33^ such as income,^34,35,36^ education,^36,37,38^ and employment.^39,40,41,42^ Existing population-level studies are limited, however, by their focus on single risk factors or on single cancer types; their use of death records unadjusted for garbage codes; and their reliance on data registries that omit certain geographic regions. In contrast, we used a death record data set that applied novel small area estimates and garbage code redistribution techniques to a full sample of US counties. We also used a series of models to explain the county disparities by identifying the most important possible mediators.

Health risk behaviors of smoking, obesity, and physical inactivity were the strongest possible mediators of the cancer disparities. This finding is consistent with prior work showing that health risk behaviors are the factors that correlate most strongly with health outcomes.^43^ With regard to cancer outcomes, there is strong evidence in particular for causal pathways that include smoking,^7^ along with evidence that rates of obesity and physical inactivity are strongly associated with cancer death rates.^8,9,10,44^ Thus, there is strong evidence to support community intervention targeting health risk behaviors. At the same time, there is a need to disrupt external factors that contribute to health risk behaviors in low- and medium-income areas. For example, efforts to increase rates of physical activity at the population level are likely to be inadequate in the absence of efforts to improve the built environments in certain communities.^45^

A second major finding was that food insecurity and the quality of clinical care may be the strongest individual mediators. There are multiple ways in which these 2 factors may account for disparities in cancer deaths. For example, low-quality clinical care may lead to delays in the diagnosis and treatment of cancer, and food insecurity may increase the incidence of certain cancers in populations due to poor nutrition, even if obesity rates are similar. Efforts to target nonbehavioral mediators might be useful in light of evidence that addressing health risk behaviors is necessary but not sufficient if the ultimate goal is to eliminate health disparities.^46,47^ In addition, the issue of regular access to healthy foods, or food security, might warrant further consideration in future studies of cancer disparities. This is because studies suggest that food insecurity is correlated with poor health,^48^ high costs,^49^ and obesity—a key risk factor for cancer.^10^ In addition, because obesity is a risk factor for diabetes and cardiovascular disease, efforts to address it might lead to substantial gains in population-level health outcomes.

To inform local health policies, it is important to generate rigorous evidence about the factors associated with cancer deaths. This is because there is a clear need for efficacy and efficiency in health programs that often are funded locally with limited budgets. Locoregional programs might be particularly helpful in addressing the clustering of cancer deaths in the South and in Appalachia. Because we identified multiple possible mediators, our findings may be useful for local policymakers to develop and evaluate future actions to eliminate cancer disparities.

Finally, to address disparities, it may be critical to maintain policies that are associated with better outcomes in low-income communities. For example, efforts to limit the expansion of Medicaid may undermine efforts to lessen socioeconomic cancer disparities, in part because the states with vs without Medicaid expansions have had larger improvements in screenings for and early detections of cancer.^50,51^ Our study adds to these findings by suggesting that limited access to affordable care is a mediator of cancer disparities, at the same time as many of the low-income counties with the highest mortality rates are in the states that eschewed the expansion. We also found that a disproportionate number of residents were non-Hispanic black Americans in low-income counties with high rates of cancer death. This highlights the need to dismantle structural racism, which contributes to inequalities in social and economic power and to the segregation of black Americans into area hot spots of counties having disproportionately more cancer deaths.

### Limitations

Our study has limitations. First, population studies are prone to interpretive errors, including ecological fallacies. For this reason, the findings from this population-level study should be used to inform population-level, but not individual-level, health programs. Second, because we studied cancer death rates in aggregate, we did not assess for differences in key factors across cancer types, which can be the focus of future work. Third, we were not able to assess for causality or for unmeasured confounding because of the cross-sectional nature of the study. Fourth, although mixed-effects models are used in the literature to assess the effects of single mediators,^52^ we were unable to find a validated approach to using a mixed-effect model in a study of multiple mediators. In part for this reason, we used a fixed-effects model (eFigure 3 in the Supplement), which means that we cannot exclude the possibility that random effects at the state level might alter the model. Fifth, we did not assess city- or county-level regulations, which limited our assessment of local health policies. Sixth, mediator models may oversimplify associations because of assumptions about directionality and interactions. For this reason, they should be used to generate rather than to confirm hypotheses for causal pathways.

## Conclusions

We found substantial gaps in cancer death rates among low-, medium-, and high-income counties. We explained much of the disparity with health behaviors, clinical care factors, health policies, and health environments. Because of the unacceptable gaps in cancer outcomes across counties that persist despite major advances in cancer care, there is an urgent need for actions to determine whether targeting these factors might ameliorate disparities.

## Notes

Supplement. **eTable 1.** Variables We Evaluated for Testing in Models of Mediation **eFigure 1.** Calculating the Disparity Risk Index **eTable 2.** Missing Values **eFigure 2.** Geographic Distributions (Maps) of “Hot Spots” Using Spatial Autocorrelation Analysis of Data for the Exposure (Median Income), Outcome (Cancer Death Rate), and Possible Mediating Factors **eFigure 3.** Fixed Effects vs Mixed Effects Models of the Possible Mediators
