# Supplementary material for: Factors Associated With Cancer Disparities Among Low-, Medium-, and High-Income US Counties
Source: JAMA Netw Open. 2018 Oct 5;1(6):e183146. doi: 10.1001/jamanetworkopen.2018.3146 (PMC6324449; doi:10.1001/jamanetworkopen.2018.3146)
Supplement: Supplement. — eTable 1. Variables We Evaluated for Testing in Models of Mediation eFigure 1. Calculating the Disparity Risk Index eTable 2. Missing Values eFigure 2. Geographic Distributions (Maps) of “Hot Spots” Using Spatial Autocorrelation Analysis of Data for the Exposure (Median Income), Outcome (Cancer Death Rate), and Possible Mediating Factors eFigure 3. Fixed Effects vs Mixed Effects Models of the Possible Mediators [file jamanetwopen-1-e183146-s001.pdf]

Supplementary Online Content

O'Connor JM, Sedghi T, Dhodapkar M, Kane MJ, Gross CP. Factors Associated With Cancer Disparities Among Low-, Medium-, and High-Income US Counties. *JAMA Netw Open.* 2018;1(6):e183146. doi:10.1001/jamanetworkopen.2018.3146

eTable 1. Variables We Evaluated for Testing in Models of Mediation

eFigure 1. Calculating the Disparity Risk Index

eTable 2. Missing Values

eFigure 2. Geographic Distributions (Maps) of "Hot Spots" Using Spatial Autocorrelation Analysis of Data for the Exposure (Median Income), Outcome (Cancer Death Rate), and Possible Mediating Factors

eFigure 3. Fixed Effects vs Mixed Effects Models of the Possible Mediators

This supplementary material has been provided by the authors to give readers additional information about their work.

**eTable 1.** Variables we evaluated for testing in models of mediation.			
**Variable**	**Description**	**Source**	**Date**
**Primary Dependent Variable**			
Cancer death rate	Age-adjusted number of deaths per 100,000 persons per year at the county level due to any cancer including benign or malignant lesions.	Institute for Health Metrics and Evaluation (IHME). United States Cancer Mortality Rates by County 1980-2014. Seattle, WA.	2014
**Primary Independent Variable**			
Median household income	Median level of income for a household in the county.	Small Area Income and Poverty Estimates	2012
**Secondary Independent Variables**			
Domain 1: Socioeconomic Factors			
Poverty [a]	Percent of persons below the federal poverty level.	American Community Survey	2011-2015
HS Grad	Percent of persons having graduated high school.	National Center for Education Statistics	2010-2011
Some college	Percent of persons having some college education.	American Community Survey	2008-2012
Unemployment	Percent being unemployed.	Bureau of Labor Statistics	2012
Income inequality	Ratio of household income at the 80th percentile to that at the 20th percentile.	American Community Survey	2009-2013
Residential segregation	The index of dissimilarity, given as the percent of the black or white residents that would have to move to a new census tract in order for them to be distributed evenly across the county.	American Community Survey	2010-2014
Domain 2: Clinical Care Factors			
Uninsured	Percent of adults who do not have health insurance.	Small Area Health Insurance Estimates	2011

eTable 1 (cont'd). Variables we evaluated for testing in models of mediation.			
Primary care physicians	Number of primary care physicians per capita.	Area Health Resource File, including data from the American Medical Association, American Hospital Association, US Census Bureau, Centers for Medicare & Medicaid Services, Bureau of Labor Statistics, and the National Center for Health Statistics.	2011
Other primary care providers	Number of non-physician primary care providers per capita.	Centers for Medicare and Medicaid Services	2013
Low-quality care	Proportion of all hospital discharges among Medicare fee-for-service beneficiaries that might be preventable because they are attributed to ambulatory-sensitive conditions.	Medicare Claims Data and the Dartmouth Atlas of Health Care.	2011
Oncologists	Number of oncologists per capita.	American Society of Clinical Oncology	2014
Presence of a comprehensive cancer center	Presence (yes/no) of at least 1 comprehensive center in a county or adjacent county.	National Cancer Institute, Office of Cancer Centers.	2014
Mammography screening	Percent screened among women aged 67-69 years in Medicare claims.	Medicare Claims Data and the Dartmouth Atlas of Health Care.	2011
Colorectal cancer screening [ab]	Percent of those aged 50 or greater receiving fecal occult blood testing or endoscopic screening.	Behavioral Risk Factor Surveillance System (BRFSS) + National Health Interview Survey (NHIS)	2008-2010
Cervical cancer screening [ab]	Percent of those aged 18 or greater without a hysterectomy receiving a Pap smear within 3 years.	BRFSS + NHIS	2008-2010
Health care costs	Price-adjusted per-enrollee reimbursement among beneficiaries of Medicare Part A and Part B.	Dartmouth Atlas of Health Care	2011
Unaffordable care	Percent being unable to see a doctor because of costs.	BRFSS	2006-2012

**eTable 1 (cont'd).** Variables we evaluated for testing in models of mediation.			
Domain 3: Health Behaviors			
Smoking (adult)	Percent of adults who were current smokers.	National Institute of Health, with pooled data from multiple sources including BRFSS	2008-2010
Obesity (adult)	Percent of adults reporting a body mass index higher than 30 kg/m^2.	BRFSS	2010
Physical inactivity (adult)	Percent of adults reporting no leisure-time physical activity.	BRFSS	2010
Sexually transmitted infections	Number of new cases of chlamydia per capita.	National Center for HIV/AIDS, Viral Hepatitis, STD, and TB prevention	2011
HIV prevalence	Number of diagnosed cases of HIV for persons aged 13 years and older in a county per 100,000 persons.	National Center for HIV/AIDS, Viral Hepatitis, STD, and TB prevention	2010
Domain 4: Physical and Health Environment Factors			
Air pollution	Average daily density of fine particulate matter.	Centers for Disease Control and Prevention Wide-ranging Online Data for Epidemiologic Research	2011
Drinking water violations	Number of reported violations per capita.	US Environmental Protection Agency Safe Drinking Water Information System	2012-2013
Limited access to healthy foods	Percent of low income persons who report they do not live near a grocery store.	US Department of Agriculture (USDA) Food Environment Atlas	2010
Food insecurity	Percent who report they did not have a reliable source of food in the past year.	Map the Meal Gap, Community Population Survey, Bureau of Labor Statistics, and American Community Survey	2011
Food environment index	An equally-weighted sum of the measures of food insecurity and limited access to healthy foods.	USDA Food Environment Atlas, Map the Meal Gap, Community Population Survey, Bureau of Labor Statistics, and American Community Survey	2010-2011

**eTable 1 (cont'd).** Variables we evaluated for testing in models of mediation.			
Access to exercise opportunities	Percent who report living reasonably close to a location for physical activity such as a park.	OneSource Global Business Browser, Delorme map data, Esri, and US Tigerline Files	2010; 2012
Severe housing problems	Percent who report living in housing that is overcrowded or severely cost burdened or without complete kitchen or plumbing facilities.	US Department of Housing and Urban Development Comprehensive Housing Affordability Strategy data	2006-2010
Domain 5: Health Policies			
Smoke-free laws	Percent who report living in an area with any type of smoke-free law.	American Lung Association, on the basis of data from the Current Population Survey Tobacco Use Supplements (TUS-CPS)	2010-2011
Medicaid-to-Medicare fee index	The state-level ratio of payments to providers for services to beneficiaries of Medicaid vs Medicare.	Kaiser Family Foundation, on the basis of data from the Urban Institute	2012
Medicaid eligibility threshold	The state-level threshold for Medicaid eligibility among working parents of dependent children as a percent of the poverty level.	Kaiser Family Foundation	2013
State cancer mandates for health insurance payments	Number of state-level mandates for insurers to reimburse for cancer prevention, screening, diagnosis, or treatment.	National Conference of State Legislatures	2009
State cancer exceptions for health insurance payment mandates	Presence of state-level exceptions to the mandates to reimburse for cancer prevention, screening, diagnosis, or treatment.	National Conference of State Legislatures	2009

**eTable 1 (cont'd).** Variables we evaluated for testing in models of mediation.			
**Demographic Variables**			
Race/ethnicity	Percent of residents who are Non-Hispanic Black, Hispanic, Asian, Native American or Alaskan	US Census Population Estimates	2012
Sex	Percent who are female.	US Census Population Estimates	2012
Rurality	Percent who are living in a mostly rural area.	US Census Population Estimates	2010
Primary language	Percent who are Non-English proficient.	American Community Survey Estimates	2008-2012

Italics = Variables that we extracted from sources other than the Robert Wood Johnson County Health Rankings.
[a] Variables that we extracted from the State Cancer Profiles database.
[b] Variables that we extracted from modeled estimates rather than direct estimates.
Underline = Variables that were evaluated for inclusion or included in the final multiple mediator model (n=19 possible mediator variables; n=4 demographic variables; n=1 primary independent variable [median household income]; and n=1 primary dependent variable [cancer death rate]).

eFigure 1. Calculating the disparity risk index.

$$\text{Disparity Risk Index} = \sum_{i=1}^{8} \omega_i \mathrm{Z}_i \,,$$

$$\text{where } \mathrm{Z}_i = \frac{\chi_i - \mu_i}{\sigma_i} \,,$$

ω = the mediator's weight, given by its standardized beta coefficient from the multiple mediator model,

x = the mediator's value,

μ = the mediator's mean,

and σ = the mediator's standard deviation.

© 2018 O'Connor JM et al. *JAMA Network Open.*

eTable 2. Missing Values	
**Variable**	**Missing Values, n (%)**
Cancer death rate	0 (0)
Median household income	0 (0)
Physical inactivity	0 (0)
Obesity	0 (0)
Poverty	0 (0)
HS Grad	449 (14)
Some college	0 (0)
Smoking	3 (<1)
Food insecurity	0 (0)
Unemployment	1 (<1)
Income inequality	0 (0)
Severe housing problems	0 (0)
Uninsured	1 (<1)
State cancer mandates for health insurance payments	0 (0)
State cancer exceptions for health insurance payment mandates	0 (0)
Food environment index	0 (0)
Colorectal cancer screening	26 (1)
Limited access to healthy foods	0 (0)
Mammography screening	85 (3)
Smoke-free laws	0 (0)
Access to exercise opportunities	24 (1)
Primary care physicians	137 (4)
Other primary care providers	123 (4)
Medicaid eligibility threshold	0 (0)
Medicaid-to-Medicare fee index	95 (3)
Air pollution	29 (1)
Drinking water violations	55 (2)
Uninsured	1 (<1)
Cervical cancer screening	26 (1)
Presence of a comprehensive cancer center	0 (0)
Oncologists	0 (0)
Low-quality care	190 (6)
Unaffordable care	754 (24)
Sexually transmitted infections	184 (6)
HIV prevalence	816 (26)
Residential segregation	359 (11)
Rurality	0 (0)
Race/ethnicity: % Black	0 (0)
Race/ethnicity: % Native American or Alaskan	0 (0)
Race/ethnicity: % Asian	0 (0)
Race/ethnicity: % Hispanic	0 (0)
Sex	0 (0)
Primary language	0 (0)
We used multiple imputation for variables with >5% and <20% missing values. We excluded variables with >20% missing values. We did not impute values for variables with <5% missing values.	

eFigure 2. Geographic distributions (maps) of “hot spots” using spatial autocorrelation analysis of data for the exposure (median income), outcome (cancer death rate), and possible mediating factors.

Getis–Ord Spatial Autocorrelations (Hot Spots): Cancer Death Rates

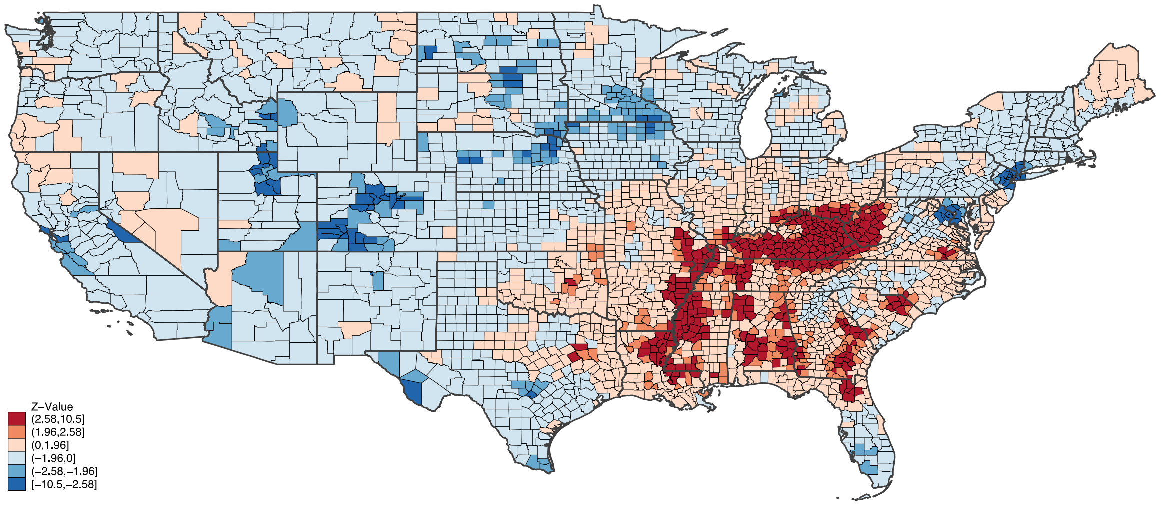

Getis-Ord G*i(d) Statistics Number of Obs = 3135

Variable	z<=-2.58	-2.58<z<=-1.96	-1.96<z<1.96	1.96<=z<2.58	2.58<=z
Cancer Death Rate	89	134	2405	179	328

Getis–Ord Spatial Autocorrelations (Hot Spots): Median Household Income

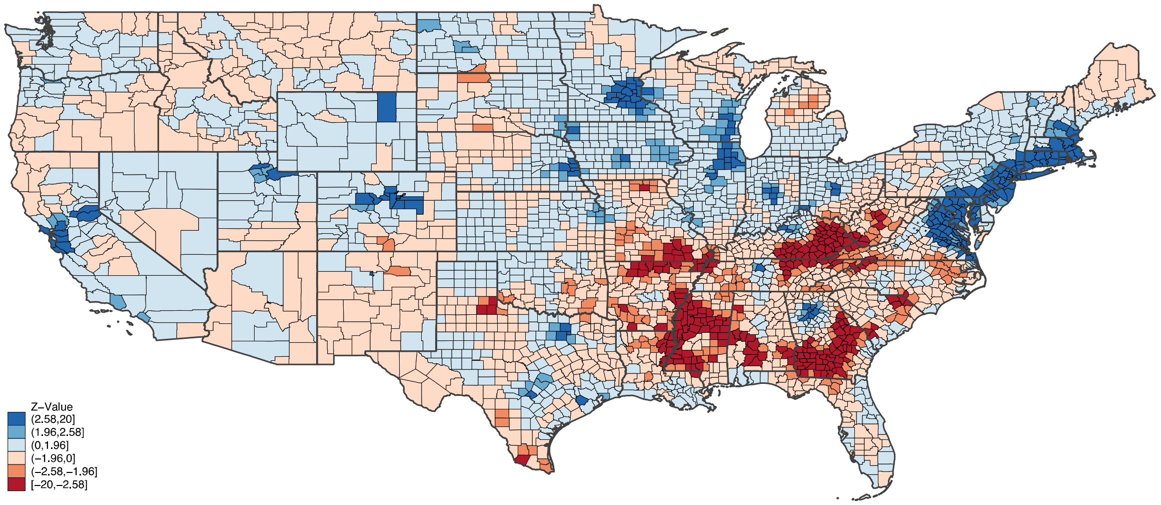

Getis-Ord G*i(d) Statistics Number of Obs = 3135

Variable	z<=-2.58	-2.58<z<=-1.96	-1.96<z<1.96	1.96<=z<2.58	2.58<=z
Median Income	244	210	2341	128	212

Getis–Ord Spatial Autocorrelations (Hot Spots): Smoking

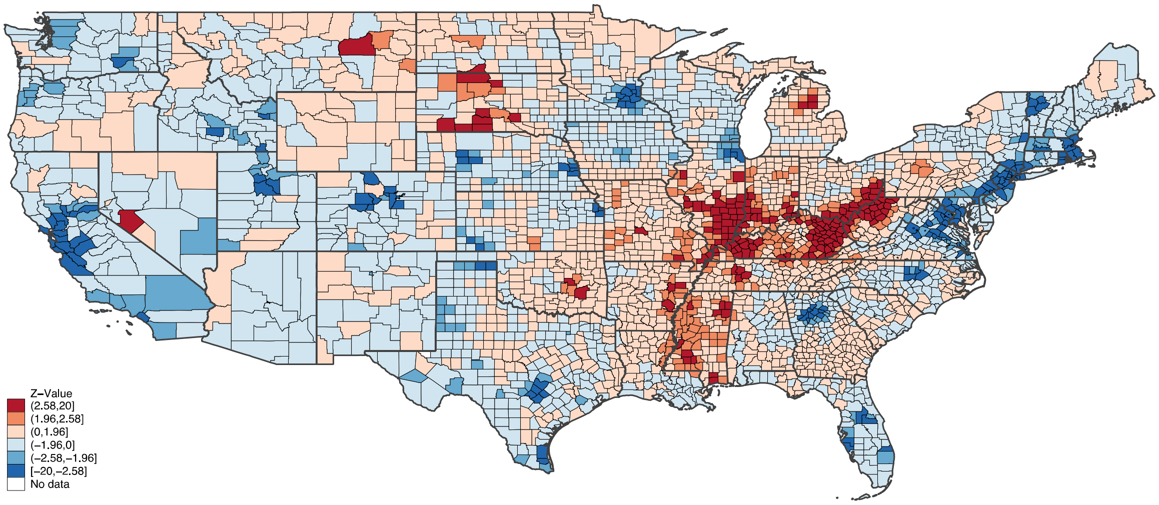

Getis-Ord G*i(d) Statistics Number of Obs = 3135

Variable	z<=-2.58	-2.58<z<=-1.96	-1.96<z<1.96	1.96<=z<2.58	2.58<=z
Smoking	171	136	2434	197	194

Getis–Ord Spatial Autocorrelations (Hot Spots): Obesity

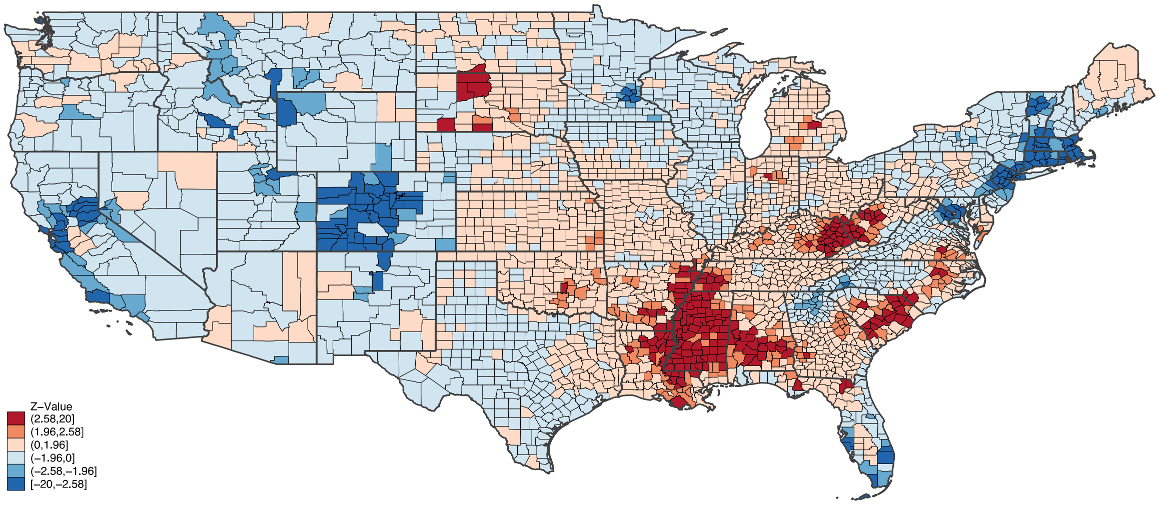

Getis-Ord G*i(d) Statistics Number of Obs = 3135

Variable	z<=-2.58	-2.58<z<=-1.96	-1.96<z<1.96	1.96<=z<2.58	2.58<=z
Obesity	143	94	2537	162	199

© 2018 O'Connor JM et al. *JAMA Network Open.*

Getis–Ord Spatial Autocorrelations (Hot Spots): Physical Inactivity

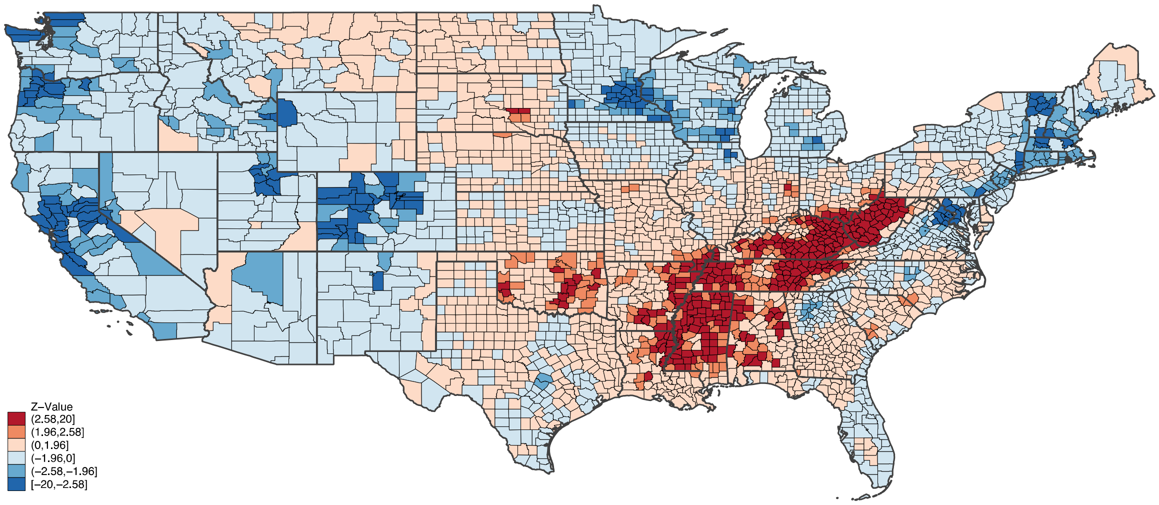

Getis-Ord G*i(d) Statistics   Number of Obs = 3135

Variable	z<=-2.58	-2.58<z<=-1.96	-1.96<z<1.96	1.96<=z<2.58	2.58<=z
Inactivity	147	172	2347	169	300

Getis–Ord Spatial Autocorrelations (Hot Spots): Food Insecurity

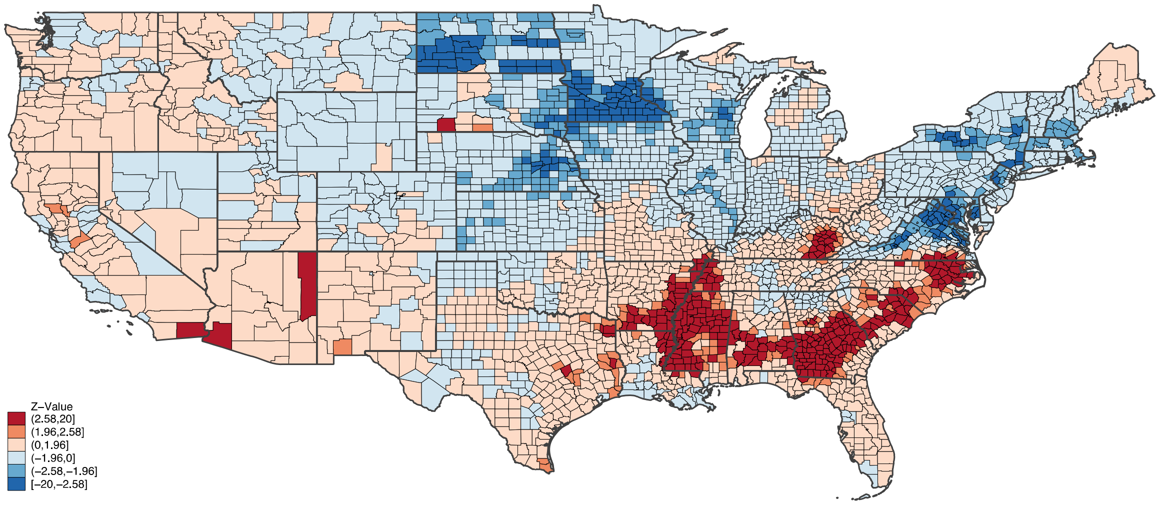

Getis-Ord G*i(d) Statistics   Number of Obs = 3135

Variable	z<=-2.58	-2.58<z<=-1.96	-1.96<z<1.96	1.96<=z<2.58	2.58<=z
Food Insecurity	157	230	2357	107	284

Getis–Ord Spatial Autocorrelations (Hot Spots): Medicaid Fee Index

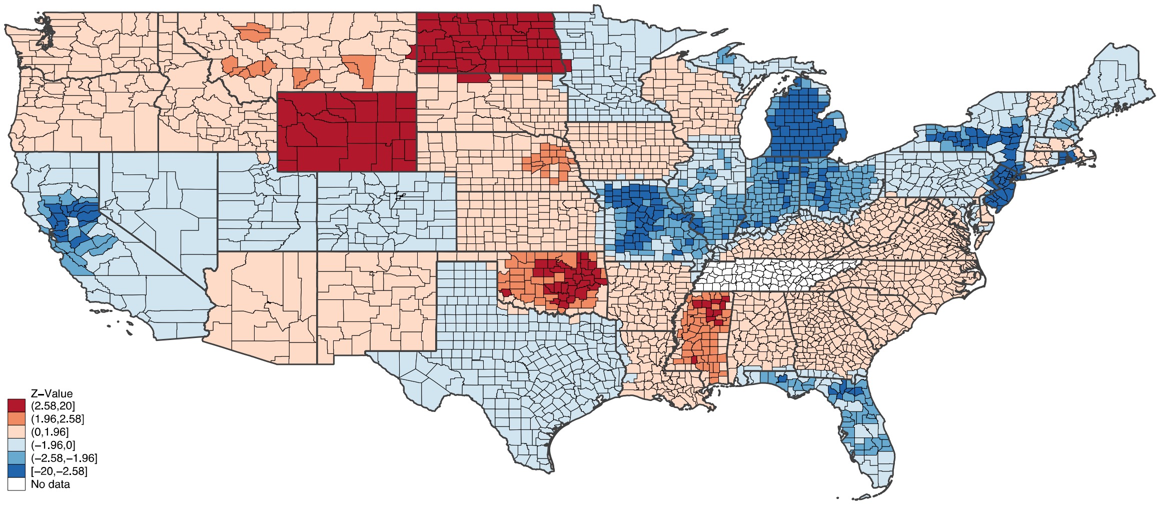

Getis-Ord G*i(d) Statistics Number of Obs = 3135

Variable	z<=-2.58	-2.58<z<=-1.96	-1.96<z<1.96	1.96<=z<2.58	2.58<=z
M. Fee Index	213	276	2296	112	143

Getis–Ord Spatial Autocorrelations (Hot Spots): Smokefree Laws

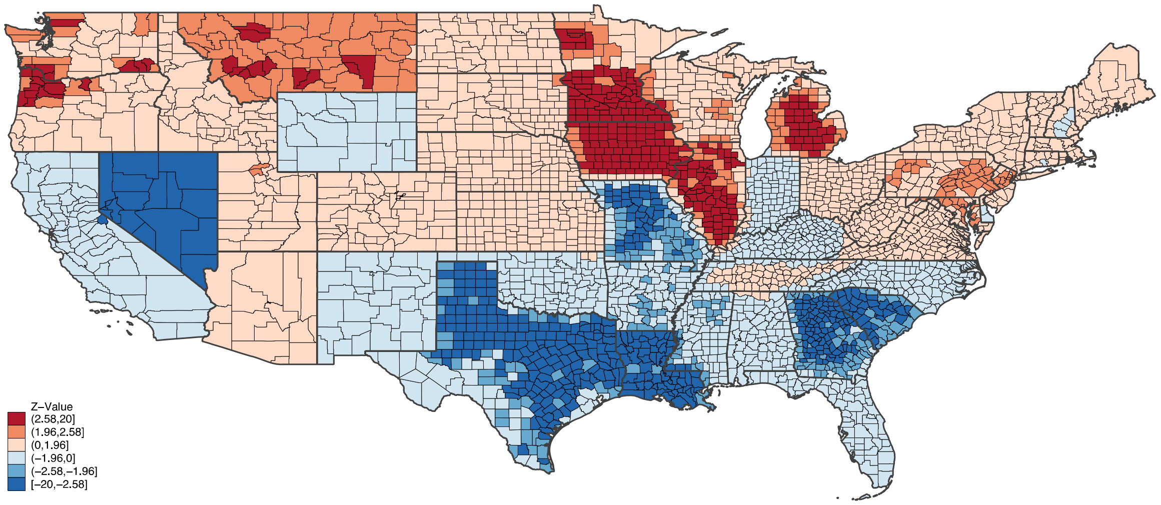

Getis-Ord G*i(d) Statistics Number of Obs = 3135

Variable	z<=-2.58	-2.58<z<=-1.96	-1.96<z<1.96	1.96<=z<2.58	2.58<=z
Smokefree	421	187	2059	201	267

Getis–Ord Spatial Autocorrelations (Hot Spots): Unaffordable Care

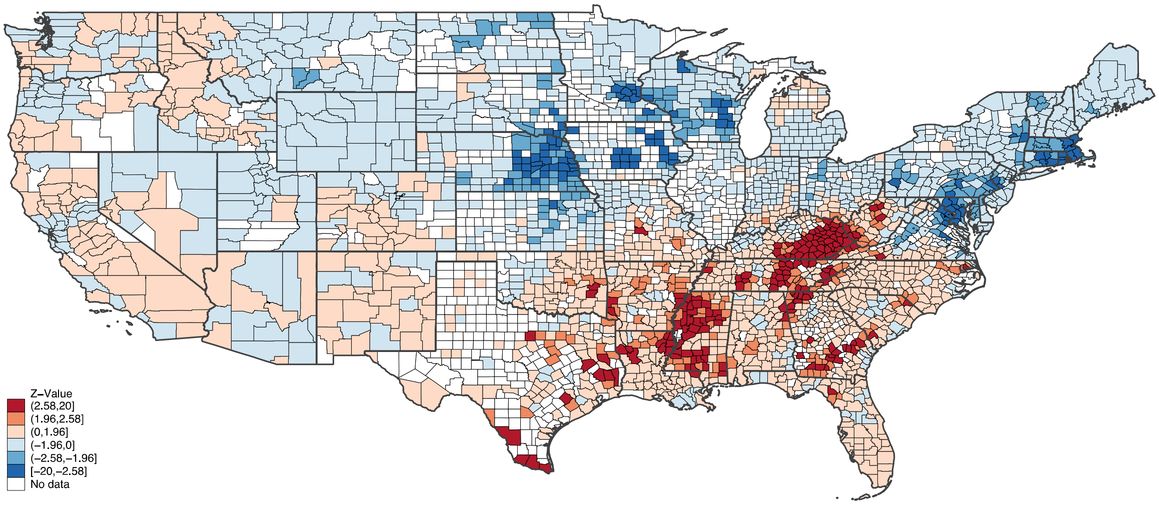

Getis-Ord G*i(d) Statistics — Number of Obs = 3135

Variable	z<=-2.58	-2.58<z<=-1.96	-1.96<z<1.96	1.96<=z<2.58	2.58<=z
Unafford. Care	108	161	1797	136	179

Getis–Ord Spatial Autocorrelations (Hot Spots): Low–Quality Care

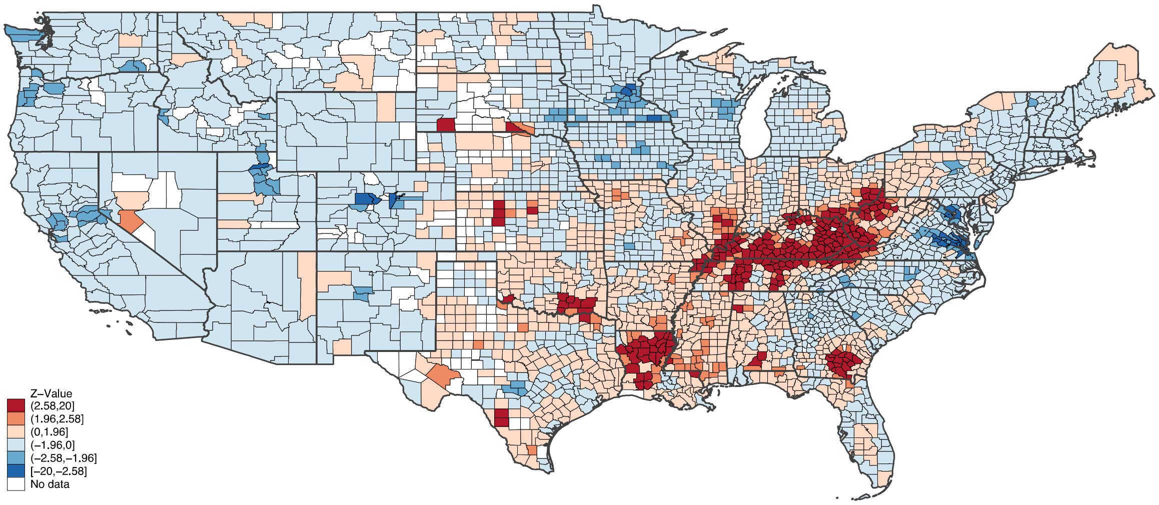

Getis-Ord G*i(d) Statistics — Number of Obs = 3135

Variable	z<=-2.58	-2.58<z<=-1.96	-1.96<z<1.96	1.96<=z<2.58	2.58<=z
Low-Qual. Care	37	109	2441	116	242

© 2018 O'Connor JM et al. *JAMA Network Open.*

eFigure 3. Fixed effects vs mixed effects models of the possible mediators.

1. Fixed effects, full model

```
Multiple-imputation estimates                  Imputations      =         20
Linear regression                              Number of obs    =      3,037

------------------------------------------------------------------------------
    mortrate |      Coef.   Std. Err.      t    P>|t|     [95% Conf. Interval]
-------------+----------------------------------------------------------------
      mhilog |  -14.05721   2.853717    -4.93   0.000    -19.65273   -8.461688
  costsnodoc |    34.9675   8.991249     3.89   0.000     17.30034    52.63466
     smoking |   .8189377   .0897163     9.13   0.000     .6430174     .994858
     obesity |   145.6288   13.05188    11.16   0.000      120.037    171.2205
  inactivity |   88.84228   12.14956     7.31   0.000     65.01879    112.6658
   smokefree |  -.3307285   .0833144    -3.97   0.000    -.4940895   -.1673674
    mcaidfee |  -14.82237   2.646088    -5.60   0.000    -20.01089   -9.633859
   foodinsec |   106.9837   18.06781     5.92   0.000     71.55502    142.4124
 preventhosp |   .2198764   .0164922    13.33   0.000     .1875003    .2522525
       rural |  -17.29486   1.486086   -11.64   0.000    -20.20876   -14.38096
      rblack |   6.811781   4.079818     1.67   0.095    -1.187797    14.81136
      rnatam |  -10.61816   5.547829    -1.91   0.056    -21.50058    .2642646
    rpacisle |   43.69257   88.64334     0.49   0.622     -130.115    217.5002
       rhisp |  -42.73555   5.081514    -8.41   0.000    -52.69927   -32.77182
      female |   51.10407   16.92414     3.02   0.003     17.91949    84.28864
   noenglish |  -83.12678   21.88129    -3.80   0.000    -126.0318   -40.22173
       _cons |   256.0453   36.99717     6.92   0.000     183.5016     328.589
------------------------------------------------------------------------------
```

2. Mixed effects, full model

```
Multiple-imputation estimates                  Imputations      =         20
Mixed-effects ML regression                    Number of obs    =      3,037

------------------------------------------------------------------------------
    mortrate |      Coef.   Std. Err.      t    P>|t|     [95% Conf. Interval]
-------------+----------------------------------------------------------------
      mhilog |  -11.52021    3.03284    -3.80   0.000    -17.46447   -5.575942
  costsnodoc |   11.32178   8.483205     1.33   0.182    -5.330002    27.97356
     smoking |   .7905099   .0878685     9.00   0.000     .6182892    .9627307
     obesity |   150.7991   14.06849    10.72   0.000     123.2253    178.3729
  inactivity |   104.0849   13.00892     8.00   0.000     78.58779     129.582
   smokefree |  -.4295455   .2606371    -1.65   0.099    -.9403849    .0812938
    mcaidfee |  -18.50561   8.031128    -2.30   0.021    -34.24633   -2.764887
   foodinsec |   143.4952   23.03619     6.23   0.000     98.34479    188.6457
 preventhosp |   .1500451   .0158498     9.47   0.000     .1189576    .1811326
       rural |   -16.0287   1.462998   -10.96   0.000    -18.89613   -13.16127
      rblack |  -9.372155   5.389709    -1.74   0.082    -19.93582    1.191513
      rnatam |  -3.525437    5.68941    -0.62   0.536    -14.67766    7.626782
      rasian |  -98.84754   19.02778    -5.19   0.000    -136.1414   -61.55363
    rpacisle |   272.7324   123.5776     2.21   0.027     30.52474      514.94
       rhisp |  -28.45702   5.915616    -4.81   0.000    -40.05145   -16.86259
      female |   25.38427   16.03025     1.58   0.113    -6.034525    56.80307
   noenglish |  -106.4828   22.30951    -4.77   0.000    -150.2089   -62.75671
       _cons |   252.4424    44.1455     5.72   0.000     165.9188    338.9661
------------------------------------------------------------------------------

------------------------------------------------------------------------------
  Random-effects Parameters  |   Estimate   Std. Err.     [95% Conf. Interval]
-----------------------------+------------------------------------------------
stateid: Identity            |
                  sd(_cons) |   9.278075   1.079277      7.386545    11.65398
-----------------------------+------------------------------------------------
               sd(Residual) |   17.63459   .2289893      17.19144    18.08916
------------------------------------------------------------------------------
```

3. Fixed effects, minus 2 state-level variables

Multiple-imputation estimates — Imputations = 20

Linear regression — Number of obs = 3,132

mortrate	Coef.	Std. Err.	t	P>|t|	[95% Conf.	Interval]
mhilog	-10.10566	2.889607	-3.50	0.000	-15.7715	-4.439808
costsnodoc	39.28835	8.882969	4.42	0.000	21.82854	56.74816
smoking	.8252575	.0896176	9.21	0.000	.6495308	1.000984
obesity	139.8433	13.0498	10.72	0.000	114.2559	165.4307
inactivity	94.68399	11.38303	8.32	0.000	72.36328	117.0047
foodinsec	143.4216	17.81976	8.05	0.000	108.4779	178.3653
preventhosp	.2317567	.0160857	14.41	0.000	.2001787	.2633348
rural	-18.00525	1.496834	-12.03	0.000	-20.9402	-15.0703
rblack	3.687628	3.941858	0.94	0.350	-4.041385	11.41664
rnatam	-21.65207	5.309147	-4.08	0.000	-32.06639	-11.23775
rasian	-39.26699	19.84936	-1.98	0.048	-78.18688	-.3471043
rpacisle	177.659	103.2778	1.72	0.085	-24.84137	380.1593
rhisp	-40.70658	5.015467	-8.12	0.000	-50.54072	-30.87243
female	49.43357	16.80095	2.94	0.003	16.49092	82.37622
noenglish	-70.11869	22.81019	-3.07	0.002	-114.8446	-25.39277
_cons	170.5315	35.03096	4.87	0.000	101.8438	239.2192
